# Genome-wide association study and genomic prediction of root system architecture in a diverse field-grown maize panel

**DOI:** 10.3389/fpls.2026.1918922

**Published:** 2026-09-15

**Authors:** Huairen Zhang, Zhigang Liu, Juan Li, Juan Liu, Jie Zang, Xiaorong Li, Guo Chen, Bo Li, Zhijie Yu, Zumuremu Tursun, Huabang Chen, Darun Cai, Xunji Chen

**Affiliations:** 1 Institute of Genetics and Developmental Biology, Chinese Academy of Sciences, Beijing, China; 2 Institute of Biological Breeding, Biological Breeding Laboratory of Xinjiang Uygur Autonomous Region Academy of Agricultural Sciences / Xinjiang Key Laboratory of Crop Biotechnology, Urumqi, China

**Keywords:** maize, root system architecture, genome-wide association study, QTN-by-environment interaction, genomic selectione, candidate gene

## Abstract

Root system architecture (RSA) plays a pivotal role in nutrient acquisition, water uptake, lodging resistance, and environmental adaptation in maize (*Zea mays* L.). However, the genetic basis of RSA at the mature stage under field conditions remains poorly characterized, primarily owing to the challenges of high-throughput phenotyping. In this study, RSA-related traits were evaluated in a diverse maize association panel across four field environments using Shovelomics combined with manual measurements. Genome-wide association analysis (GWAS) was performed using the BLINK and 3VmrMLM models, identifying four main-effect loci consistently detected by both methods and three additional loci exhibiting significant QTN-by-environment interactions (QEIs).Notably, SNP AX-90556586 on chromosome 9 was consistently associated with nodal and crown root number across environments. Its candidate gene encodes a leucine-rich repeat receptor-like serine/threonine-protein kinase with similarity to BRI1/SOBIR1-like proteins, implicating hormone-mediated root development. Genomic prediction revealed that marker subsets enriched for significant loci enhanced within-population prediction, whereas genome-wide markers better captured the polygenic architecture required for robust cross-population prediction. These findings provide insights into the genetic architecture and environmental responsiveness of maize RSA and offer genomic resources for improving root traits in breeding programs.

## 1 Introduction

Root system architecture (RSA), defined as the spatial configuration of root components in the soil, plays a fundamental role in plant anchorage, nutrient acquisition, water uptake, and adaptation to heterogeneous soil environments (Lynch, 1995). In maize, RSA is closely associated with agronomically important traits, including drought and flooding tolerance, lodging resistance, nutrient use efficiency, and yield stability under suboptimal conditions (Giuliani et al., 2005; Cai et al., 2012; Saengwilai et al., 2014). The functional efficiency of a root system depends not only on individual root traits such as number, angle, density, and branching pattern, but also on the coordinated development and spatial distribution of different root classes. Understanding the genetic basis of RSA is therefore essential for improving maize adaptation to diverse environments and for developing root ideotypes with enhanced resource acquisition and stress resilience.

Maize (*Zea mays* L.) has a complex root system consisting of embryonic and post-embryonic root components. The embryonic root system includes the primary root and seminal roots, whereas the post-embryonic root system comprises crown roots, brace roots, and lateral roots, which dominate the mature root architecture and contribute substantially to soil exploration and mechanical support (Gao and Lynch, 2016; Hochholdinger et al., 2018). Previous studies using mutants have identified several genes involved in maize root development, including genes controlling primary root elongation, seminal root initiation, lateral root formation, and root hair development (Wen et al., 2005; Woll et al., 2005; Bray and Topp, 2018). However, RSA traits are typically polygenic, environmentally sensitive, and developmentally dynamic. Their expression often depends on complex interactions among genotype, developmental stage, and field environment (Burton et al., 2015; Zurek et al., 2015; Ju et al., 2018; Schneider et al., 2020).

Despite the importance of RSA, genetic dissection of field-grown mature maize root systems remains challenging. Root traits are difficult to phenotype because they are hidden belowground, labor-intensive to sample, and sensitive to soil conditions. Consequently, previous studies have focused on seedling root traits under controlled conditions (Pace et al., 2015), paper-roll (Abdel-Ghani et al., 2013), or greenhouse environments (Zhang et al., 2025). Although these approaches have provided valuable insights into early root development, they may not capture the genetic regulation of mature root systems under field conditions. Field-based phenotyping methods, such as Shovelomics and manual enumeration, offer a promising approach to overcome these limitations.

Genome-wide association studies (GWAS) exploit natural variation within diverse germplasm panels to dissect the genetic architecture of such complex traits (Wang and Zhou, 2017; Xiao et al., 2017; Tibbs Cortes et al., 2021; Zeng et al., 2022). While early root GWAS were primarily conducted under controlled seedling conditions, recent methodological advances (2024–2025) have accelerated the transition toward large-scale field investigations. For instance, recent studies have leveraged multi-view imaging and multivariate GWAS frameworks to capture comprehensive root structures from over 1,700 field-grown root crowns (Hein et al., 2025), and have integrated micro-phenotypic root slice traits to pinpoint over 3,500 RSA-associated loci, including *ZmFUT5* (Li et al., 2025).

Despite these advances, several knowledge gaps remain. First, most maize root GWAS have focused on the seedling stage under controlled conditions, which may not fully reflect the genetic regulation of mature root systems in the field conditions (Pace et al., 2015; Zhang et al., 2025). Second, few studies have systematically evaluated the environmental plasticity of RSA traits and its genetic basis across multiple field environments (Schneider et al., 2020). Third, the utility of GWAS-derived markers for genomic prediction of field-grown root traits has not been thoroughly assessed (Ren et al., 2022; Sha et al., 2023).

In this study, we analyzed a diverse maize association panel of 368 inbred lines genotyped with the Axiom™ Maize 600K SNP Array. RSA-related traits were evaluated across four field environments using Shovelomics and manual enumeration. The objectives were to: (i) characterize the genetic diversity, population structure, and linkage disequilibrium of the association panel; (ii) evaluate natural variation and environmental plasticity of mature-stage maize RSA traits; (iii) identify significant loci and candidate genes associated with RSA traits through GWAS; and (iv) assess whether GWAS-associated markers can improve genomic prediction accuracy for RSA traits. This study provides genetic resources and candidate loci for understanding maize RSA and supports root-focused molecular breeding strategies.

## 2 Materials and methods

### 2.1 Plant materials and field experiments

A diverse maize association panel comprising 368 inbred lines was used in this study. The panel was genotyped using the Axiom™ Maize 600K SNP Array and phenotyped for RSA-related traits under field conditions. Field trials were conducted in a single-replication design across four environments representing distinct location–year combinations: Hainan 2017 (HN_2017), Xinjiang 2019 (XJ_2019), Xinjiang 2020 (XJ_2020), and Xinjiang 2021 (XJ_2021). In each environment, all inbred lines in the association panel were planted once per replication and arranged in a randomized order within the field.

For each line, three plants exhibiting uniform growth without obvious disease symptoms, mechanical damage, or severe lodging were selected for root sampling. Root phenotyping was performed at the physiological maturity to capture field-grown adult root architecture. Detailed information on locations, planting density, soil conditions, and experimental design is provided in **Supplementary Table 1**.

### 2.2 RSA phenotyping

Root system architecture traits were measured using Shovelomics (Trachsel et al., 2011) combined with manual enumeration. The procedure consisted of the following steps:

1. Plant selection: At physiological maturity, three representative plants per line showing uniform growth and free from obvious disease symptoms, mechanical damage, or severe lodging were selected for root sampling.
2. Root crown excavation: A shovel was inserted approximately 25–30 cm from the plant stem at a depth of ~30 cm to excavate the root crown while preserving the major root components (brace roots, crown roots, and seminal roots). The soil monolith containing the root system was carefully lifted from the ground.
3. Soil removal and washing: Adhering soil was gently removed by shaking, followed by washing with low-pressure water to expose the root crown structure. Care was taken to avoid damage to fine roots during washing.
4. Trait measurement: The following nine RSA traits were measured immediately after washing:

1. Root angle (Angle): the angle of brace roots relative to the soil surface, measured with a protractor (degrees).
2. Root density (Density): visually scored density of roots within the root crown using a 1–5 scale, where 1 indicates very sparse root distribution and 5 indicates very dense root distribution.
3. Brace root whorls (BRW): the number of visible brace root whorls above the soil surface.
4. Number of first-whorl brace roots (N1stBR): the number of brace roots on the topmost whorl.
5. Number of second-whorl brace roots (N2ndBR): the number of brace roots on the second whorl from the top.
6. Number of remaining brace roots (NLBR): the total number of brace roots on additional lower whorls.
7. Number of crown roots (NCR): the total number of crown roots below the soil surface.
8. Number of nodal/seminal roots (NSR): the total number of nodal roots, including seminal roots.
9. Total root number (TR): the sum of all counted brace, crown, and seminal roots.

5. All measurements were performed by the same trained personnel across environments to minimize operator bias.

### 2.3 Statistical analysis of phenotypic data

#### 2.3.1 Descriptive statistics and ANOVA

Phenotypic variation was characterized using descriptive statistics, including mean, range, standard deviation, coefficient of variation (CV), skewness, and kurtosis. Prior to analysis of variance (ANOVA), Levene’s test (Levene et al., 1960) implemented in the R package car (Fox and Weisberg, 2019) was performed to assess the homogeneity of variances across environment groups. For all traits where the homogeneity assumption was violated at the significance level of *P* < 0.05, Welch’s one-way ANOVA was adopted to evaluate environmental effects, followed by the Games-Howell test for *post-hoc* multiple comparisons among different environments for all RSA-related traits.

#### 2.3.2 Variance components and heritability

To estimate variance components and broad-sense heritability, a linear mixed model (LMM) was fitted in which genotype and environment were treated as random effects:


$${\text{Y}}_{\text{ij}}=\text{μ}+{\text{G}}_{\text{i }}+{\text{E}}_{\text{j}}+{\text{ϵ}}_{\text{ij}}$$

Where ${\text{Y}}_{\text{ij}}$ represents the phenotypic value of the (i)-th inbred line in the (j)-th environment, μ is the overall mean, ${\text{G}}_{\text{i}}$ is the random effect of genotype (inbred line), ${\text{E}}_{\text{j}}$ is the random effect of environment (Loc_year), and ${\text{ϵ}}_{\text{ij}}$ is the residual error. The model was fitted using the lmer() function in the R package lme4 (version: 2.0.1; Bates et al., 2015): lmer(value ~ (1|inbred_line) + (1|Loc_year), data = data). Therefore, broad-sense heritability (*H^2^*) across environments was obtained from the above LMM. Best linear unbiased predictions (BLUPs) were obtained as random effects of genotypes from the above LMM, and used for Pearson correlation analysis among traits.

#### 2.3.3 Environmental plasticity

Environmental plasticity of each RSA trait was quantified using the coefficient of variation (CV) across the four environments for each inbred line (Schlichting, 1986; Wang and Zhou, 2017). For a given inbred line i and trait t, the phenotypic plasticity was calculated as CV_(i, t)_= SD_(i, t)_/Mean_(i, t)_; where SD_(i, t)_ is the standard deviation and Mean_(i,t)_is the arithmetic mean of trait t for inbred line i across the four environments (HN_2017, XJ_2019, XJ_2020, XJ_2021). The CV-based phenotypic plasticity was chosen for its intuitive interpretation as a standardized, scale-free metric that facilitates comparison across traits with different units and magnitudes (Schlichting, 1986). However, the CV-based plasticity has limitations: it becomes unstable when trait means approach zero, and it cannot partition variation into genetic and non-genetic components. For the root count and angle traits measured here—all with non-zero, positive means—CV remains an interpretable metric of environmental responsiveness.

### 2.4 Genotyping, population structure, and LD decay

Genomic DNA was extracted from young leaves of each inbred line and genotyped using the Axiom™ Maize 600K SNP Array. SNPs were filtered by marker quality, minor allele frequency (MAF ≥ 0.05), and missing rate (≤ 10%). After quality control, 465,112 high-quality SNPs were retained for subsequent analyses.

Population structure was assessed using STRUCTURE v2.3.4 (Pritchard et al., 2000) and Principal Component Analysis (PCA) was calculated by TASSEL 5 (Bradbury et al., 2007). The optimal number of subpopulations (K) was determined using the Evanno method implemented in STRUCTURE HARVESTER (Earl and vonHoldt, 2011).

Whole genome linkage disequilibrium (LD) decay was estimated by PLINK 1.07 with parameters “Plink --noweb --bfile input_file --r2 --ld-window-kb 1000 --ld-window-r2 0 --ld-window 10000 --out ld.txt” (Purcell et al., 2007). The average LD decay distance was defined as the physical distance at which r^2^ declined to half of its maximum value.

### 2.5 Genome-wide association study

Genome-wide association studies (GWAS) for RSA traits and environmental plasticity were performed using the Bayesian-information and Linkage-disequilibrium Iteratively Nested Keyway (BLINK) model (Huang et al., 2019). This multi-locus approach utilized two fixed-effect models iteratively to increase statistical power and computational efficiency. The significance threshold for associations was established using Bonferroni correction based on the total number of markers (*P* = 0.05/465112). Manhattan and quantile-quantile (Q-Q) plots were generated to visualize significant associations and evaluate the control of population structure.

To complement the BLINK analysis and to evaluate the robustness of associations across environments, the 3VmrMLM model (Li et al., 2022a) implemented in R package IIIVmrMLM (Li et al., 2022b) was applied as an alternative multi-environment GWAS approach. Marker-trait associations were identified using the package’s default significance criteria, which included both significant (SIG) and suggestive (SUG) associations. Significant SNPs repeatedly detected across environments, traits, or environmental plasticity indices were considered stable or pleiotropic loci. The phenotypic variance explained (PVE) by each significant SNP was estimated as the adjusted r^2^ from a single-marker linear regression model.

### 2.6 Genome-wide G×E interaction analysis

To identify SNPs exhibiting significant genotype-by-environment interactions (G×E), the 3VmrMLM model (Li et al., 2022a) was applied using the R package IIIVmrMLM (Li et al., 2022b). Unlike single-environment GWAS models, 3VmrMLM model jointly used multi-environment phenotypic data by fitting random SNP effects across environments through a three-variance-component multi-locus mixed model. This approach simultaneously tests for quantitative trait nucleotides (QTNs) main effects and QTN-by-environment interactions (QEIs). Significant QEIs were identified using the package’s default significance criteria, which included both significant (SIG) and suggestive (SUG) associations.

### 2.7 Candidate gene identification and functional annotation

Candidate genes were identified within the upstream and downstream regions of each lead SNP based on the average LD decay distance. Candidate genes were prioritized according to the following criteria: physical distance to the lead SNP, LD support, functional annotation, expression in root-related tissues, involvement in hormone signaling or stress response, and evidence from previous studies.

### 2.8 Trait-associated haplotype analysis

Haplotype analysis was performed using all significant SNPs associated with specific trait because the SNP density of the genotyping array was insufficient to define local haplotypes around individual association loci. Lines with missing genotypes were excluded from haplotype comparisons. Haplotypes represented by a sufficient number of lines were retained for statistical analysis, whereas rare haplotypes with very small sample sizes were excluded. Phenotypic differences among haplotypes were tested by Kruskal-Wallis rank sum test.

### 2.9 Genomic prediction and model evaluation

Genomic prediction was performed using ridge regression BLUP (rrBLUP) (Endelman, 2011). Prediction accuracy was calculated as the Pearson correlation between observed and predicted phenotypic values. Five-fold cross-validation method was performed by randomly partitioning the panel into training (80%) and testing (20%) sets, repeated 5 times. To compare different marker sets, prediction models were built using: (i) genome-wide SNPs after quality control; (ii) GWAS-associated SNPs. An independent set of 156 maize inbred lines was used for external validation. The validation population was curated from a diverse panel of inbred lines.

## 3 Results

### 3.1 Population structure and linkage disequilibrium (LD) decay of the association panel

Population structure analysis using STRUCTURE identified seven major subpopulations, which were further validated using representative inbred lines from known heterotic groups (**Figure 1A**, **Supplementary Figure S1**). Subpopulation 1 consisted of 32 inbred lines and was classified as the Lancaster I group, with OH43 serving as the representative line. Subpopulation 2 corresponded to the Reid group and was represented by PH6WC. Subpopulation 3 was assigned to the Lv da Red Cob group, represented by Dan3130. Subpopulation 4, which contained the largest number of inbred lines, was considered the Lancaster II group, with PH4CV as its representative line. The remaining subpopulations corresponded to the P, SPT, and Improved Reid groups, represented by P178, Ji853, and Zheng58, respectively.

**Figure 1:**
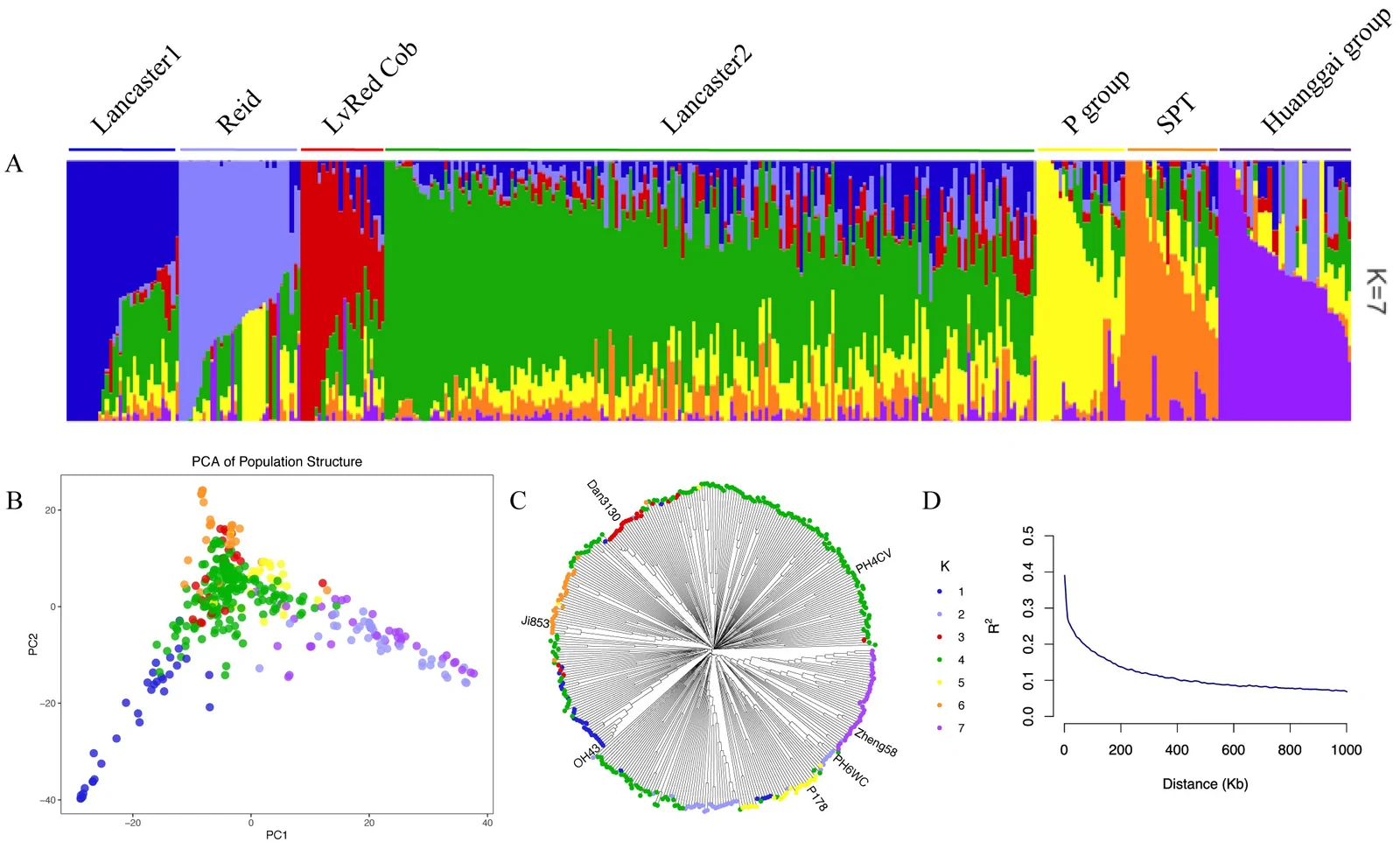
The population structure of the novel association mapping panel. **(A)**: Population structure of the novel association panel using STRUCTURE software. Each vertical column represents one inbred line and each colored segment in each column represents the proportion contributed from ancestral populations. **(B)**: PCA of the novel association panel, individuals from the same group divided by STRUCTURE are represented by the corresponding color. **(C)**: Phylogenetic tree of the novel association mapping population; representative inbred lines from each K cluster from STRUCTURE were visualized. **(D)**: LD decay of the association panel on whole genome.

To further validate the major patterns of population structure, Principal Component Analysis (PCA) and phylogenetic analysis were performed. The results were jointly visualized with the STRUCTURE clustering pattern, and representative lines from each STRUCTURE-defined subpopulation were annotated on the phylogenetic tree. Overall, the clustering patterns inferred from STRUCTURE, PCA (**Figure 1B**), and phylogenetic analysis (**Figure 1C**) were largely consistent, with only a small number of inbred lines showing discrepancies among methods. Linkage disequilibrium (LD) decayed rapidly with increasing physical distance (**Figure 1D**). The average LD decay distance was less than 100 kb, indicating extensive historical recombination during maize evolution and breeding.

### 3.2 Phenotypic variation and environmental plasticity of RSA traits

The RSA traits were evaluated under field conditions by Shovelomics and manual enumeration across four environments (**Figure 2A**). Substantial phenotypic variation was observed for all nine RSA traits(**Supplementary Table 2**). The brace root whorls (BRW) ranged from 2.83 to 5.92, with an average of 4.41. The Angle varied from 28.78 to 74.71, yielding a mean of 45.46. Density ranged from 0.92 to 4.08. The brace root trait encompassed the number of roots in the first whorls (N1stBR), second whorls (N2ndBR), and additional whorls (NLBR), with N1stBR displaying a range of 9.25 to 33.42, indicating substantial variability among the maize inbred lines. Similar patterns were noted for the other root traits, with total roots (TR) exhibiting the highest standard deviation (SD) and BRW the lowest. Heritability estimates ranged from 0.24 for NCR to 0.71 for TR, indicating that some root traits are more genetically stable than others (**Supplementary Figure S2**).

**Figure 2:**
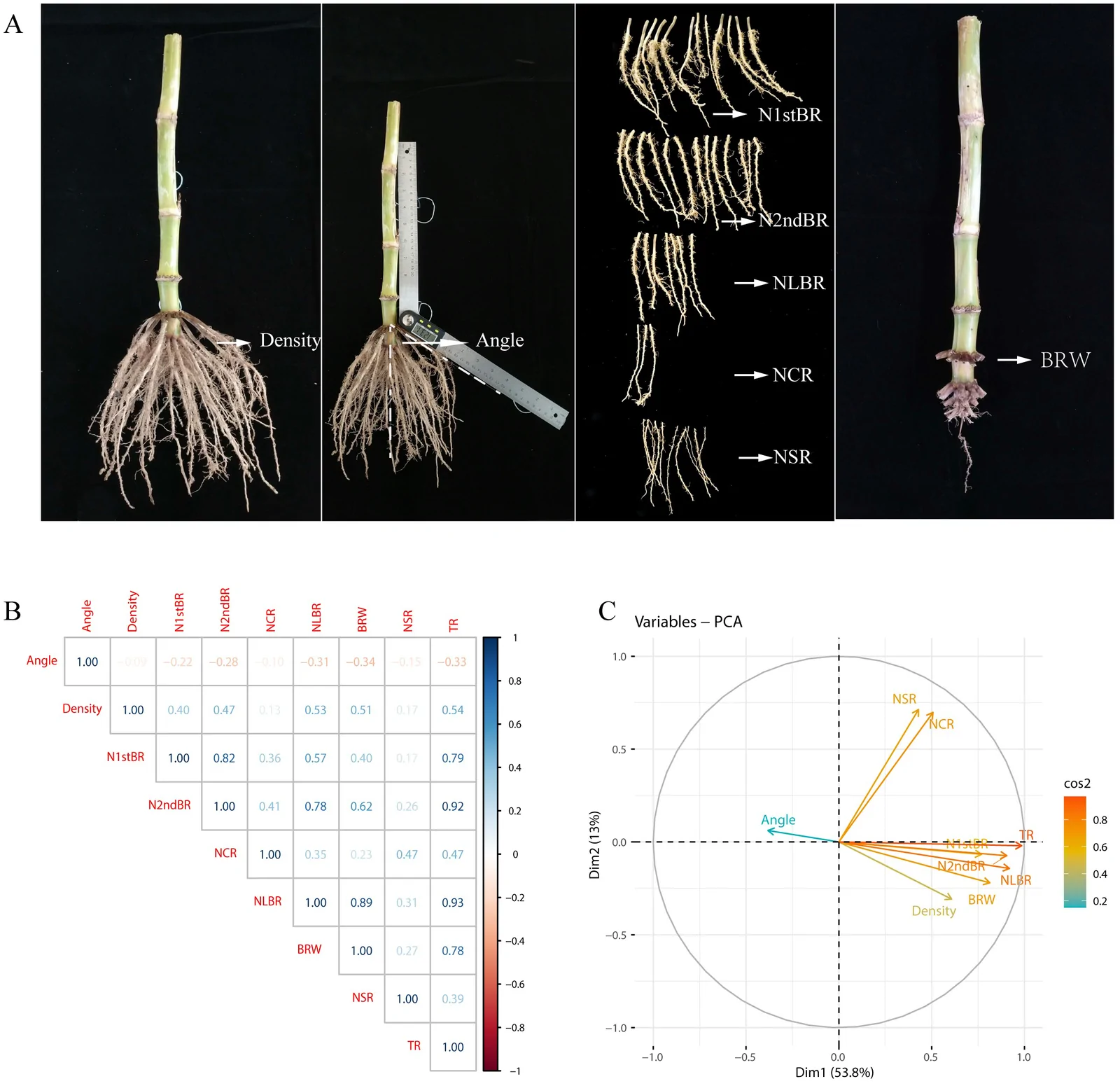
The RSA traits investigation, correlation and principle component analysis. **(A)**: Dissection of maize RSA traits using Shovelomics method, density (Density, visual score from 1 to 5), angle (Angle, measured by protractor), brace root whorls (BRW, manually count), number of brace root on the top whorl (N1stBR, anually count), number of brace root on the second whorl (N2ndBR, anually count), number of the left brace root on other whorls (NLBR, anually count), number of crown roots (NCR, anually count), number of seminal roots (NSR, anually count), number of total roots (TR, sum of all brace root, crown roots and seminal roots). **(B)**: Correlation coefficient matrix between nine RSA traits, heatmap bar shows the correlation coefficients ranged -1 to 1, tests with no significant were not shown in box. **(C)**: PCA of variables, heatmap of bar shows the size of square cosine, which is the quality of representation of the variables on factor map. a high cos2 indicates a good representation of the variable on the principal component.

Levene’s test was conducted to assess the homogeneity of variances among the four environments. The results indicated that the assumption of equal variances was violated for all nine root system architecture (RSA)-related traits (*P* < 0.05). Therefore, Welch’s one-way ANOVA was used to evaluate environmental effects. Significant differences among the four environments were detected for all nine RSA-related traits (*P* < 0.001). Games-Howell *post hoc* tests were subsequently performed to identify pairwise differences among environments. For all nine RSA-related traits, HN_2017 differed significantly from XJ_2019, XJ_2020, and XJ_2021, suggesting that differences in environmental conditions, particularly the lateritic soil at Hainan and the calcareous soil at Xinjiang, substantially influenced maize root system architecture (**Supplementary Figure S3A**). Among the four environments, total root number (TR) exhibited a clear rank order: XJ_2019 > XJ_2021 > XJ_2020 > HN_2017, with all three Xinjiang environments exceeding Hainan. This pattern was driven predominantly by post-embryonic brace roots: N1stBR was highest in XJ_2019 and lowest in HN_2017, and NLBR was significantly lower in HN_2017 than in all Xinjiang environments. In contrast, embryonic root traits showed the opposite environmental trend: both NCR and NSR were consistently higher in HN_2017 than in the three Xinjiang environments. Root angle (Angle) was lowest in XJ_2021, the environment with the most extreme summer heat, suggesting that brace roots adopted a more horizontal orientation under severe abiotic stress. The plasticity analysis of each RSA trait revealed that Density, NLBR, and NSR exhibited greater plasticity compared to the other RSA traits measured (**Supplementary Figure S3B**).

Correlation analysis showed that root number-related traits (NCR, NSR, TR) were positively correlated with each other, whereas root angle showed weak negative correlations with root number traits (**Figure 2B**). Principal Component Analysis (PCA) of the variables further confirmed the negative correlation of the angle with other RSA traits (**Figure 2C**). The Biplot analysis suggested that the first principal component could be interpreted as the post-embryonic root system, represented by N1stBR, N2ndBR, NLBR, BRW, TR, and Density, which are critical for providing nutrition and structural support to maize during later developmental stages. In contrast, the second principal component was interpreted as the embryonic root system, represented by NSR and NCR, which play essential roles in maize nutrition and anchorage during early developmental phases.

### 3.3 GWAS dissects the main-effect loci and environment-dependent genetic variations underlying maize RSA in contrasting soil environments

We first performed a genome-wide association study (GWAS) for 9 root system architecture (RSA) traits and root plasticity using the Bayesian-information and Linkage-disequilibrium Iteratively Nested Keyway (BLINK) model. At the predefined genome-wide significance threshold, a total of 66 significant SNPs were identified, which were distributed across all 10 maize chromosomes and explained 1.00%-31.53% of the phenotypic variation for corresponding traits (**Figure 3**, **Supplementary Tables 3**, **4**).

**Figure 3:**
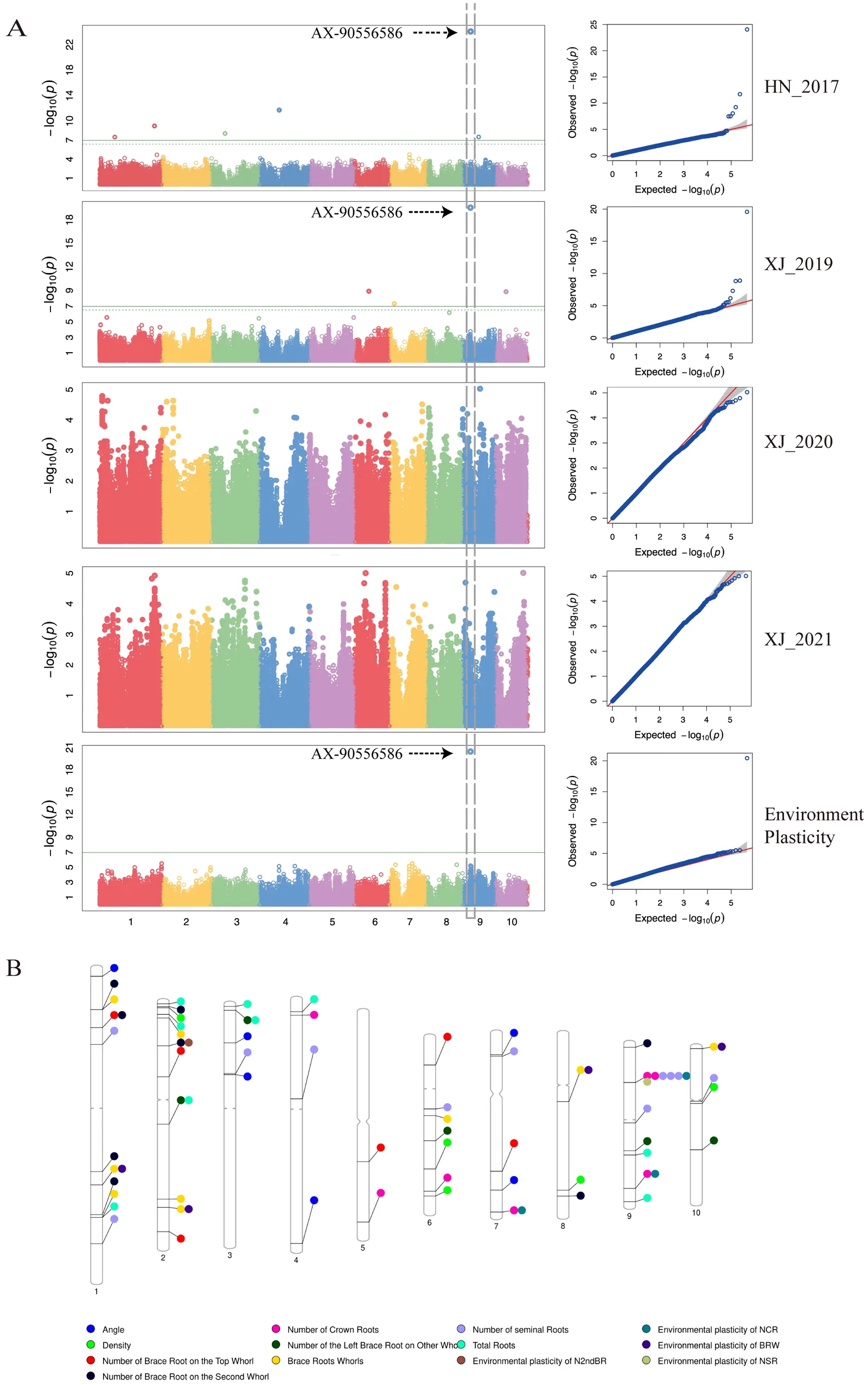
Genome wide association mapping of RSA traits within four environment and environmental plasticity by BLINK. **(A)**: Manhattan plots and Q-Q (quantile-quantile) plots of genome wide association mapping of NSR in four environments (HN_2017, XJ_2019, XJ_2020, XJ_2021) and the environmental plasticity of NSR. **(B)**: Distribution of significant SNPs associated with nine RSA traits and Environmental plasticity on maize genome. AX-90556586, a stable locus on chromosome 9 consistently associated with NCR and NSR, is highlighted in blue on the Manhattan plots.

Specifically, 20 significant loci were detected for root architecture traits, including root angle, root density and the number of visible brace root whorls above the soil surface (BWR). For brace root-related traits (N1stBR, N2ndBR, NLBR), 19 significant SNPs were identified, among which the SNP AX-90665607 was simultaneously associated with both N1stBR and N2ndBR. For crown root number (NCR), 7 significant SNPs were detected; for nodal/seminal root number (NSR), 11 significant SNPs were identified, and 9 significant SNPs were detected for total root number. Quantile-quantile (Q-Q) plots confirmed that the BLINK model adequately controlled for population stratification, ensuring the reliability of the detected associations (**Figure 3A**). Multiple loci were shared among crown root number, seminal root number and total root number, indicating a partially overlapping genetic regulatory network underlying these interconnected root traits (**Figure 3B**).

To further dissect the genetic basis of RSA in multi-environment trials, we applied the Three-Variance Multi-locus Random-SNP-effect Mixed Linear Model (3VmrMLM), to systematically identify main-effect quantitative trait nucleotides (QTNs) and QTN-by-environment interactions (QEIs) across all test environments. Using this multi-environment analysis, a total of 242 main-effect QTNs and 210 QEIs were respectively identified for all nine RSA-related traits (**Supplementary Figures S4**, **S5**).

Subsequent overlap analysis showed that 4 main-effect QTNs detected by 3VmrMLM were also identified as significant SNPs in the BLINK analysis (**Supplementary Figure S6A**). These shared loci were significantly associated with N2ndBR (AX-90761400, AX-91798419), NCR (AX-90973644), NLBR (AX-90761400) and BRW (AX-91512155), respectively. Notably, 3 QEIs identified by 3VmrMLM overlapped with the significant SNPs from BLINK (**Supplementary Figure S6B**): AX-90556586 was associated with NCR, NSR and BRW; AX-91512155 was associated with BRW; and AX-91511518 was associated with NLBR.

The three overlapping SNPs with QEI signals exhibited pronounced environment-dependent quantitative trait locus effects, where both the magnitude and direction of their genetic effects varied substantially across environments (**Supplementary Figures S7A, B**). For additive effects, AX-90556586 regulated BRW, NCR and NSR in a strictly environment-dependent manner: its effect on BRW shifted from negative in HN_2017 to positive in XJ_2019, and then turned slightly negative again in XJ_2020 and XJ_2021. For NCR and NSR, AX-90556586 showed positive effects in HN_2017, strong negative effects in XJ_2019, and reverted to positive effects in XJ_2020 and XJ_2021. AX-91511518 displayed a particularly strong additive interaction for NLBR: its effect was nearly zero in HN_2017, strongly negative in XJ_2019, and positive in XJ_2020 and XJ_2021, with the largest positive effect observed in XJ_2021. The additive effect of AX-91512155 on BRW also shifted from negative in HN_2017 and XJ_2019 to positive in XJ_2020 and XJ_2021.

Pronounced environment-specific dominance effects were also observed for these loci. The dominance effect of AX-91512155 on BRW was negative in HN_2017 and XJ_2019, but became positive in XJ_2020 and XJ_2021. Similarly, AX-91511518 showed positive dominance effects on NLBR in HN_2017, negative effects in XJ_2019 and XJ_2020, and a strong positive effect in XJ_2021. These typical crossover interaction patterns demonstrate that the genetic effects of the three SNPs are strongly dependent on environmental conditions, highlighting the substantial contribution of QEI effects to the phenotypic variation of maize root system architecture traits.

### 3.4 Candidate gene prioritization and Trait-associated haplotype analysis

The BLINK model identified 66 significant SNPs, whereas the 3VmrMLM model detected 242 QTNs and 210 QEIs. Among the main-effect loci, four SNPs were consistently identified by both BLINK and 3VmrMLM, while three additional SNPs detected by BLINK corresponded to significant QTN-by-environment interactions (QEIs) in the 3VmrMLM analysis. Notably, AX-91512155 was repeatedly detected by the 3VmrMLM model as both a main-effect QTN and a QEI. Candidate genes associated with these robust loci and their functional annotations are summarized in **Table 1**.

**Table 1:** The common loci identified by BLINK and 3VmrMLM model and their candidate gene annotation.

SNP	Chromosome	Position	Trait	Detected by different models	Candidate gene	Function annotation
AX-90761400	2	117773787	N2ndBR, NLBR	BLINK 3VmrMLM_QTN	GRMZM2G162056	hagtf13 - GNAT-transcription factor 13
AX-91798419	9	107198796	N2ndBR	BLINK 3VmrMLM_QTN	GRMZM2G172357	Grx_C4-glutaredoxin subgroup I
AX-90973644	5	202292728	NCR	BLINK 3VmrMLM_QTN	GRMZM2G164948	Thioredoxin reductase
AX-91512155	2	15686398	BRW	BLINK, 3VmrMLM_QTN 3VmrMLM_QEI	NA	NA
AX-90556586	9	37540531	NCR,NSR BRW	BLINK 3VmrMLM_QEI	GRMZM2G027958	Leucine-rich repeat receptor-like serine/threonine/tyrosine-protein
AX-91511518	2	12351954	NLBR	BLINK 3VmrMLM_QEI	GRMZM2G125669	(aox2 - alternative oxidase2)

Among these loci, AX-90556586 on chromosome 9 was of particular interest because it was repeatedly associated with NCR, NSR, and BRW. In the BLINK analysis, this locus showed highly significant associations with NSR (HN_2017: P = 3.92Í10^-19^, PVE = 15.34%; XJ_2019: P = 1.25Í10^-14^, PVE = 31.53%), NCR (P = 3.80Í10^-28^, PVE = 23.97% in XJ_2019), and the corresponding phenotypic plasticity of both traits (P = 1.72Í10^-15^, PVE = 18.66% for NSR; P = 4.70Í10^-4^, PVE = 10.44% for NCR). Consistent with these results, the 3VmrMLM analysis identified significant QTN-by-environment interactions (QEIs) for NSR, NCR, and BRW at the same locus (P = 2.68Í10^-120^, 2.92Í10^-43^, and 1.46Í10^-17^, respectively), further supporting its importance in regulating root system architecture across environments.

Regional linkage disequilibrium (LD) analysis identified GRMZM2G027958 as the most plausible candidate gene within this association interval, showing strong LD with neighboring SNPs (**Figure 4A**). This gene encodes a leucine-rich repeat (LRR) receptor-like serine/threonine protein kinase, and publicly available transcriptome data showed that it is highly expressed in primary roots at 7 days after germination (**Figure 4B**), suggesting a potential role in root development.

**Figure 4:**
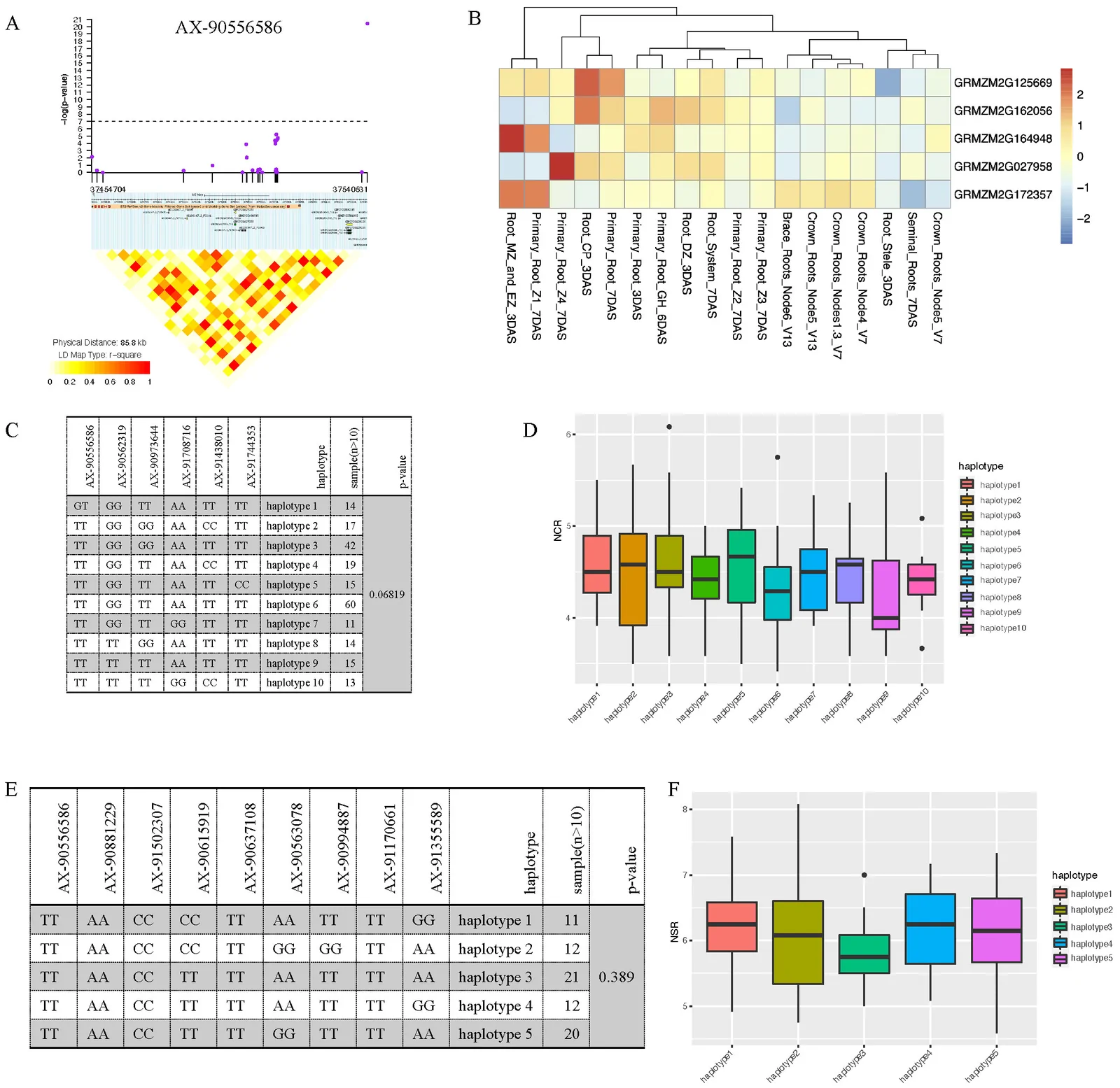
Candidate gene prioritization and haplotype analysis. **(A)** regional LD block; annotated genes from 100Kb of upstream and downstream of the lead SNP (AX-90556586) were plotted, and genes located within the corresponding interval were screenshot from the genome browser of maize reference genome B73AGP v2 to coordinate with the positions of SNPs. The linkage disequilibrium correlation coefficients (r^2^) between pair-wise markers were computed and visualized by LD heatmap using genotype data of whole population. The deeper the heatmap, the stronger LD existed between SNPs. To mark the different associations, SNP name was plotted as the title of the figure; **(B)** The heatmap of candidate gene expression identified by both BLINK and 3VmrMLM model; expression data were from inbred line B73 RNA-seq gene expression atlas. Tissues were only selected with root related tissues, names were marked by tissue names and development stages; **(C)** Haplotypes of significant SNPs for NCR; **(D)** Boxplots of phenotype of different haplotypes for NCR; **(E)** Haplotypes of significant SNPs for NSR; **(F)** Boxplots of phenotype of different haplotypes for NSR. The Kruskal-Wallis rank sum test was performed to compare the statistically phenotype differences between haplotypes; Interquartile range (IQR)s were presented on the bound of the box, with the central line denoting the median. The whiskers extend to 1.5 times the IQR, while individual data points falling outside this range are considered outliers (represented by circles).

To further evaluate the combined effects of the significant SNPs associated with NCR and NSR, haplotype analysis was performed separately for each trait. Significant phenotypic differences were observed among the haplotype groups for both NCR and NSR, enabling the identification of favorable haplotypes associated with improved trait performance (**Figures 4C–F**).

### 3.5 Genome-wide markers provide better generalization ability and prediction stability across genetically independent populations

rrBLUP model was used to genomic prediction with two marker strategies: (i) genome-wide SNP markers and (ii) GWAS-associated markers derived from all significant loci identified by BLINK, 3VmrMLM QTNs and 3VmrMLM QEIs. Prediction accuracy was evaluated in both the association panel and an independent validation population.

In the association panel, genomic prediction based on GWAS-associated markers generally achieved higher prediction accuracies than models using genome-wide SNP markers for most traits (**Figure 5A**). 3VmrMLM_QTNs has the highest prediction accuracy for all RSA traits, followed by the BLINK model. The improvement was particularly evident for TR (0.82), N2ndBR (0.77), and NLBR (0.77), suggesting that trait-associated loci detected by 3VmrMLM model captured a substantial proportion of the genetic variation underlying these traits. Across all evaluated traits, prediction accuracies based on GWAS-associated markers were moderate to high, indicating that the significant loci identified through GWAS provided effective predictive information within the discovery population.

**Figure 5:**
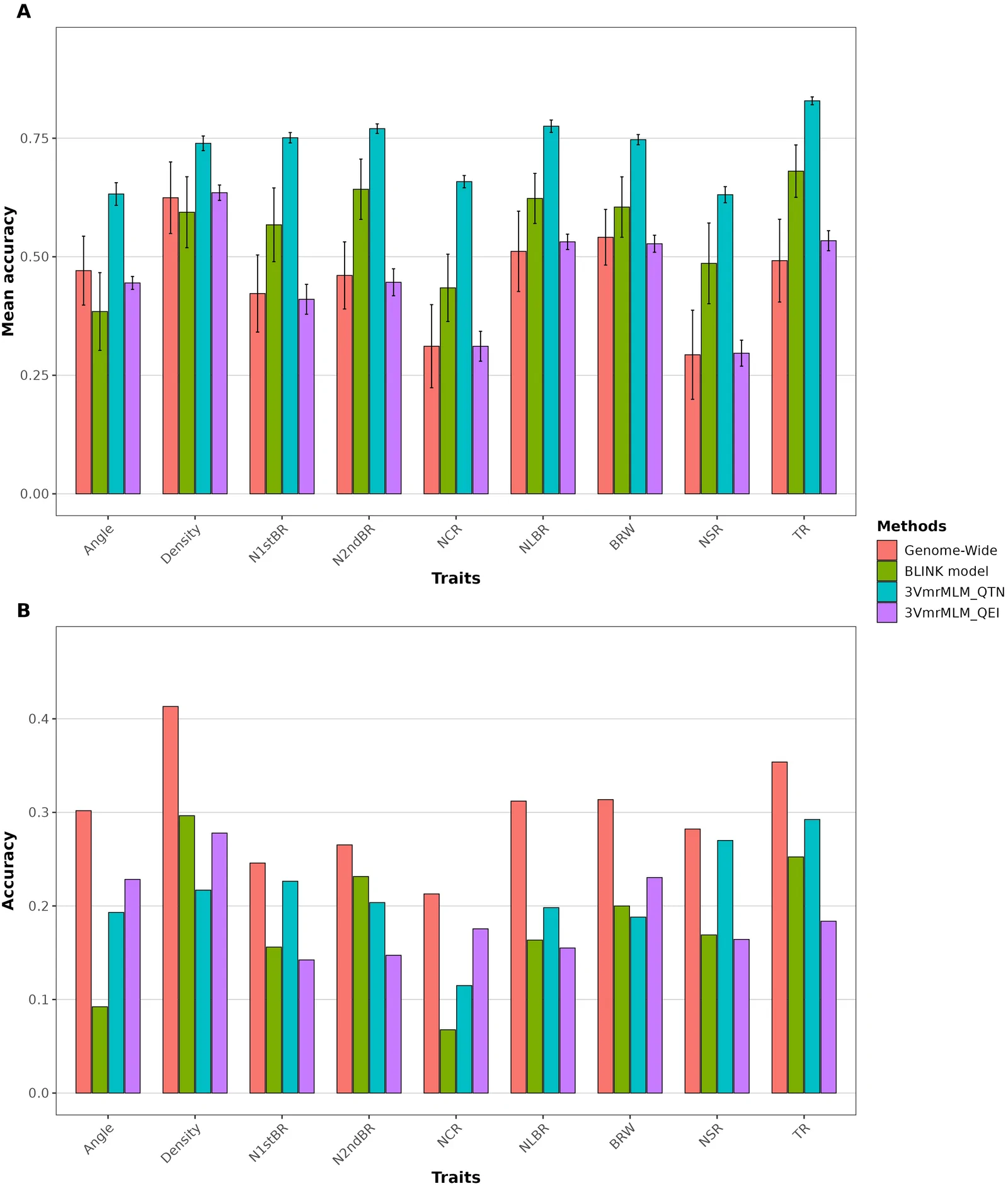
Prediction accuracy of two strategies, strategy I: genome wide SNPs, and strategy II: Three GWAS associated significant SNPs (BLINK model, 3VmrMLM_QTN, 3VmrMLM_QEI). **(A)** Prediction accuracy on association population; **(B)** Prediction accuracy on an independent validation population. BRW: brace root whorls; N1stBR: number of brace root on the top whorl, N2ndBR: number of brace root on the second whorl; NLBR: number of the remaining brace root on other whorls; NCR, number of crown roots; NSR, number of nodal/seminal roots; TR, total number of brace roots, crown roots and seminal roots. Error bars were represented by mean ± standard deviation.

To further evaluate the robustness of the prediction models, an independent validation population consisting of 156 individuals was genotyped and phenotyped separately for all studied traits. In contrast to the results obtained in the association panel, genomic prediction using genome-wide SNP markers consistently outperformed prediction based on all GWAS-associated markers(BLINK, 3VmrMLM_QTNs, 3VmrMLM_QEIs) across nearly all traits in the independent population (**Figure 5B**). Prediction accuracies obtained using GWAS-associated markers decreased substantially in the validation population, whereas models using genome-wide markers maintained relatively stable predictive performance.

These results indicate that GWAS-derived markers can improve prediction accuracy within the training population; however, genome-wide markers provide better generalization ability and prediction stability across genetically independent populations.

## 4 Discussion

### 4.1 A field-based association panel provides a valuable resource for maize RSA research

RSA is a key determinant of water uptake, nutrient acquisition, lodging resistance, and environmental adaptation. However, mature root systems remain difficult to phenotype under field conditions. In this study, we evaluated 368 diverse maize inbred lines for nine RSA traits across four field environments using Shovelomics and manual enumeration. The panel exhibited abundant genetic and phenotypic diversity, moderate population structure, and rapid LD decay (~100 kb), features that are well-suited for GWAS and candidate gene discovery.

Compared with previous studies that focused on seedling traits under controlled conditions (Pace et al., 2015; Abdel-Ghani et al., 2013; Song et al., 2016; Zhang et al., 2025), this study provides field-based genetic information for mature-stage RSA. Adult root systems — including crown roots, brace roots, and lateral roots — contribute substantially to soil exploration, mechanical support, and stress adaptation. The phenotypic and genotypic datasets generated here thus represent useful resources for the maize RSA research community.

### 4.2 The genetic architecture of maize RSA is complex and environment-dependent

BLINK model identified 66 significant SNPs corresponding to 55 candidate genes. Only a small number of loci were detected across multiple environments, whereas most were environment-specific or associated with environmental plasticity, indicating strong genotype-by-environment interactions for mature-stage RSA.

The comparison between BLINK and 3VmrMLM further highlights the complexity of the genetic architecture underlying maize RSA. BLINK is effective for identifying loci with relatively stable marginal effects within a single environment, and therefore performs well for detecting major-effect SNPs in environment-specific GWAS. By contrast, 3VmrMLM jointly models phenotypic variation across environments and can detect both main-effect QTNs and QTN-by-environment interactions (QEIs), making it more suitable for dissecting loci whose effects are environmentally dependent. In the present study, only a small subset of loci was consistently detected across methods, whereas several loci resolved by 3VmrMLM showed clear environment-responsive effects. Therefore, the apparent inconsistency between single-environment GWAS and multi-environment GWAS should not be interpreted as contradiction, but rather as evidence that a substantial proportion of RSA loci are sensitive to environmental context.

The limited overlap between candidate genes identified here and those in previous studies further underscores the complexity of RSA genetics. Only two candidate genes (GRMZM2G075701 and GRMZM2G125934) overlapped with the study by Ren et al. (2022), one gene (GRMZM2G040736) overlapped with Wu (Wu et al., 2022), and one gene (GRMZM2G017557) matched previously registered root-related genes in MaizeGDB. These differences likely reflect variation in germplasm composition, developmental stage, phenotyping method, trait definition, and statistical models. Notably, this study used manually counted traits (root number), whereas previous studies often extracted image-based topological features (root angle, width, projected area) (Pace et al., 2015; Song et al., 2016; Zhang et al., 2025). Such methodological differences can lead to distinct associated loci, which does not imply inconsistency but rather complementary genetic information. The PVE of individual SNPs ranged from minor to moderate, with several loci exceeding 20%. The cumulative PVE did not fully explain observed heritability, indicating missing heritability attributable to small-effect loci, rare variants, epistasis, structural variants, and G×E interactions.

### 4.3 Environmental determinants of RSA genetic variation

The contrasting RSA responses across environments were biologically interpretable. Variance analysis showed that field conditions affected not only the overall magnitude of RSA traits but also the allocation pattern among root classes. Total root number (TR) was consistently higher in the Xinjiang environments than in HN_2017, reaching its maximum in XJ_2019, indicating that the arid inland environments favored greater overall root production. This increase was mainly driven by post-embryonic brace-root traits, as first-whorl brace roots (N1stBR) and lower brace roots (NLBR) were significantly more abundant in Xinjiang and consistently exceeded the values observed in HN_2017. By contrast, crown roots (NCR) and nodal/seminal roots (NSR), which are embryonic in origin and contribute primarily to early nutrient acquisition and anchorage, were consistently more abundant under the humid, acidic sandy loam conditions of Hainan (HN_2017). Thus, the contrast between HN_2017 and the Xinjiang sites did not simply increase or decrease RSA uniformly, but instead shifted the developmental balance between embryonic and post-embryonic root classes. In addition, the lower root angle observed in XJ_2021 suggests that extreme summer heat may have promoted a more horizontal brace-root configuration, further supporting the idea that field environments reshape RSA through coordinated changes in multiple component traits.

### 4.4 AX-90556586 and GRMZM2G027958 represent a promising receptor-like kinase locus for root number traits

AX-90556586 on chromosome 9 was the most promising locus identified in this study, as it was repeatedly associated with NSR, NCR, and environmental plasticity, with PVE values reaching 31.53%. In the single-environment BLINK analysis, this locus was strongly associated with NCR and NSR in HN_2017 and XJ_2019, but it was not detected as a significant locus in XJ_2020 or XJ_2021. By contrast, 3VmrMLM identified AX-90556586 as a significant QEI, although not as a significant main-effect QTN, indicating that its genetic effect was strongly environment dependent rather than consistently expressed across all field conditions. This difference between BLINK and 3VmrMLM is biologically and statistically reasonable. BLINK evaluates marginal SNP effects within a single environment, whereas 3VmrMLM jointly models phenotypic variation across environments and is therefore better able to capture loci whose effects vary with environmental context.

The environment-dependent behavior of AX-90556586 provides a clear example of how field conditions modulate genotype effects on RSA. Multi-environment analysis showed that the additive effects of this locus were not constant across environments. For NCR and NSR, the effect was positive in HN_2017, strongly negative in XJ_2019, and became positive again in XJ_2020 and XJ_2021, indicating a typical crossover interaction pattern. These results suggest that the same allele can promote or reduce root-number traits depending on environmental context. Therefore, the absence of a significant BLINK signal in XJ_2020 and XJ_2021 should not be interpreted as evidence of no biological effect, but rather as evidence that the phenotypic expression of this locus was strongly conditioned by local field environments. This interpretation is consistent with the climatic contrast among the test sites, because extreme warmth in 2020 and exceptional summer heat in 2021 may have altered phenotypic expression and reduced the power to detect stable marginal SNP effects in single-environment GWAS. This limitation is likely exacerbated by the low minor allele frequency (MAF = 0.08) of AX-90556586.

The high PVE estimates for AX-90556586 should therefore be interpreted with caution. In GWAS, low-frequency variants can yield inflated PVE estimates for several reasons. First, under an additive model, the variance attributed to a SNP is influenced by its allele frequency, and rare variants inherently contribute less to population-level phenotypic variance despite potentially large per-allele effects (Wray et al., 2013). Second, when one allele class is represented by a relatively small number of individuals, individual phenotypic extremes can disproportionately influence the regression slope and the resulting r² estimate (Yang et al., 2011). Third, unbalanced allele frequencies reduce the effective sample size for estimating genotype-class means, leading to larger standard errors and potentially inflated effect-size estimates due to the Beavis effect at moderate sample sizes. The higher PVE observed in XJ_2019 than in HN_2017 may therefore partly reflect environmental differences in phenotypic variance rather than a true increase in genetic effect size. Independent validation in larger and more genetically balanced populations will be necessary to obtain less biased effect-size estimates and to evaluate the breeding value of this locus more accurately.

The candidate gene GRMZM2G027958 encodes an LRR receptor-like kinase with BRI1/SOBIR1-like annotations (Cruz et al., 2020). Brassinosteroids regulate cell division, elongation, vascular development, and root meristem activity (Manghwar et al., 2022), and brassinosteroid signaling has been implicated in root elongation, lateral root development, and environmental responses (Lanctot, 2022; Wei and Li, 2016). A BRI1/SOBIR1-like receptor kinase located near a stable environment-responsive SNP associated with root number traits is therefore biologically plausible as a regulator of maize RSA. Although the association evidence is strong, the causal relationship between GRMZM2G027958 and RSA variation remains to be validated experimentally. Future work should include root tissue-specific expression profiling, hormone-response assays, CRISPR/Cas9 mutagenesis, and complementation experiments.

### 4.5 GWAS-informed genomic prediction: promise and limitations

The comparison between genome-wide SNP markers and GWAS-associated markers revealed distinct advantages and limitations of the two genomic prediction strategies. In the association panel, prediction models based on GWAS-associated markers generally achieved higher accuracies than models using genome-wide markers. This result is likely because the selected GWAS-associated loci were directly linked to trait-associated genetic variation identified within the same population. By enriching markers with major-effect loci, the model can reduce background noise from non-informative markers and improve prediction efficiency within the training population.

However, the prediction performance of GWAS-associated markers decreased markedly in the independent validation population. In contrast, genome-wide marker-based prediction maintained superior and more stable accuracies across traits. This discrepancy may reflect population-specific effects of GWAS loci. Significant SNPs identified in the association panel may be strongly associated with traits due to linkage disequilibrium patterns, allele frequencies, or genetic backgrounds unique to that population. When applied to an independent population, these associations may weaken because of recombination, altered LD structure, or differences in genetic architecture (Rabier et al., 2016).

Genome-wide marker approaches capture both major and minor genetic effects distributed across the entire genome and therefore better represent the polygenic nature of complex root traits. As a result, whole-genome marker models generally exhibit stronger transferability and robustness across populations. Similar observations have been reported in previous genomic selection studies, where marker subsets derived from GWAS improved within-population prediction but often showed reduced predictive ability across unrelated populations (Habier et al., 2007; Wray et al., 2013).

Together, these findings suggest that GWAS-associated markers may be useful for population-specific prediction and marker-assisted breeding, whereas genome-wide marker-based genomic selection is more suitable for broad application across diverse breeding populations.

### 4.6 Limitations and future perspectives

Several limitations of this study should be acknowledged to guide future research. First, candidate genes were identified based on physical proximity to lead SNPs within LD intervals (mean LD decay ~100 kb), supported by functional annotations and publicly available root expression profiles. However, direct functional evidence — such as gene expression data from root tissues of the association panel itself, mutant phenotypes, CRISPR/Cas9 knockout or knockdown lines, or transgenic complementation — is currently lacking. Although trait-level haplotype analysis was performed using GWAS-associated SNPs, gene-based haplotype analysis for this candidate gene could not be conducted because the SNP density of the current genotyping array was insufficient to define local haplotypes within the associated genomic region. Second, multi-omics integration (e.g., root tissue-specific transcriptomics, proteomics, and metabolomics of the same panel) would substantially strengthen the evidence linking candidate genes to root developmental phenotypes. Third, although this study evaluated RSA across four field environments, replication within each environment was limited to a single replicate, which may reduce statistical power to detect G×E interactions and to estimate environment-specific genetic effects.

Future work should prioritize: (i) functional validation of the most promising candidates, particularly GRMZM2G027958, using reverse genetics approaches (CRISPR/Cas9 mutagenesis, overexpression), candidate gene based haplotype analysis and tissue-specific expression profiling across root developmental stages; (ii) multi-omics characterization of root tissues from the association panel across developmental stages and environments to identify expression quantitative trait loci (eQTLs) and protein-level variation associated with RSA; and (iii) replicated multi-environment field trials with controlled stress gradients (water deficit, nitrogen limitation, soil compaction) to dissect G×E interactions at higher resolution and to develop predictive models for root ideotype performance under specific environmental scenarios.

## 5 Conclusion

This study characterized the natural variation, environmental plasticity, and genetic architecture of mature-stage RSA in 368 diverse maize inbred lines across four field environments. GWAS was performed using the BLINK and 3VmrMLM models, identifying four main-effect loci consistently detected by both methods and three additional loci exhibiting significant QTN-by-environment interactions (QEIs). Among these, AX-90556586 on chromosome 9 was repeatedly associated with nodal and crown root traits, and its candidate gene GRMZM2G027958, encoding an LRR receptor-like kinase with BRI1/SOBIR1-like features, represents a biologically plausible target for functional validation. GWAS-associated markers improved within-population prediction accuracy, supporting their utility in marker-assisted breeding, whereas genome-wide markers provided greater robustness across diverse breeding populations. These findings provide genetic resources for understanding maize RSA and for developing root-focused molecular breeding strategies.

## Data Availability

The names of the repository/repositories and accession number(s) can be found in the article/**Supplementary Material**.

## References

[B1] Abdel-Ghani A. H. Kumar B. Reyes-Matamoros J. Gonzalez-Portilla P. J. Jansen C. Martin J. P. S. . (2013). Genotypic variation and relationships between seedling and adult plant traits in maize (*Zea mays* L.) inbred lines grown under contrasting nitrogen levels. Euphytica 189, 123–133. doi: 10.1007/s10681-012-0759-030311153

[B2] Bates D. Mächler M. Bolker B. Walker S. (2015). Fitting linear mixed-effects models Usinglme4. J. Stat. Software 67, 1–48. doi: 10.18637/jss.v067.i01

[B3] Bradbury P. J. Zhang Z. Kroon D. E. Casstevens T. M. Ramdoss Y. Buckler E. S. (2007). TASSEL: Software for association mapping of complex traits in diverse samples. Bioinformatics 23, 2633–2635. doi: 10.1093/bioinformatics/btm308 17586829

[B4] Bray A. L. Topp C. N. (2018). The quantitative genetic control of root architecture in maize. Plant Cell Physiol. 59, 1919–1930. doi: 10.1093/pcp/pcy141 30020530 PMC6178961

[B5] Burton A. L. Johnson J. Foerster J. Hanlon M. T. Kaeppler S. M. Lynch J. P. . (2015). QTL mapping and phenotypic variation of root anatomical traits in maize (*Zea mays* L.). Theor. Appl. Genet. 128, 93–106. doi: 10.1007/s00122-014-2414-8 25326723

[B6] Cai H. Chen F. Mi G. Zhang F. Maurer H. P. Liu W. . (2012). Mapping QTLs for root system architecture of maize (*Zea mays* L.) in the field at different developmental stages. Theor. Appl. Genet. 125, 1313–1324. doi: 10.1007/s00122-012-1915-6 22718302

[B7] Cruz D. F. De Meyer S. Ampe J. Sprenger H. Herman D. Van Hautegem T. . (2020). Using single‐plant‐omics in the field to link maize genes to functions and phenotypes. Mol. Syst. Biol. 16, MSB20209667. doi: 10.15252/msb.20209667 33346944 PMC7751767

[B8] Earl D. A. vonHoldt B. M. (2011). STRUCTURE HARVESTER: A website and program for visualizing STRUCTURE output and implementing the evanno method. Conserv. Genet. Resour. 4, 359–361. doi: 10.1007/s12686-011-9548-730311153

[B9] Endelman J. B. (2011). Ridge regression and other kernels for genomic selection with R package rrBLUP. Plant Genome 4, 250–255. doi: 10.3835/plantgenome2011.08.0024

[B10] Fox J. Weisberg S. (2019). An R Companion to Applied Regression. 3rd ed (Thousand Oaks, CA: Sage).

[B11] Gao Y. Lynch J. P. (2016). Reduced crown root number improves water acquisition under water deficit stress in maize (*Zea mays* L.). J. Exp. Bot. 67, 4545–4557. doi: 10.1093/jxb/erw243 27401910 PMC4973737

[B12] Giuliani S. Sanguineti M. C. Tuberosa R. Bellotti M. Salvi S. Landi P. (2005). Root-ABA1, a major constitutive QTL, affects maize root architecture and leaf ABA concentration at different water regimes. J. Exp. Bot. 56, 3061–3070. doi: 10.1093/jxb/eri303 16246858

[B13] Guo W. J. Wang F. H. Lv J. Y. Yu J. Wu Y. Wuriyanghan H. . (2025). Phenotyping, genome-wide dissection, and prediction of maize root system architecture. iMeta 4, e70015. doi: 10.1002/imt2.70015 PMC11995184 40236777

[B14] Habier D. Fernando R. L. Dekkers J. C. M. (2007). The impact of genetic relationship information on genome-assisted breeding values. Genetics 177, 2389–2397. doi: 10.1534/genetics.107.081190 18073436 PMC2219482

[B15] Hein K. M. Liu A. E. Mullen J. L. Shao M.-R. Topp C. N. McKay J. K. (2025). Phenome-to-genome insights for evaluating root system architecture in field studies of maize. Plant Genome 18, e70100. doi: 10.1002/tpg2.70100 40851219 PMC12375851

[B16] Hochholdinger F. Marcon C. Baldauf J. A. Yu P. Frey F. P. (2018). Proteomics of maize root development. Front. Plant Sci. 9, 143. doi: 10.3389/fpls.2018.00143 29556242 PMC5844967

[B17] Huang M. Liu X. Zhou Y. Summers R. M. Zhang Z. (2019). BLINK: a package for the next level of genome-wide association studies with both individuals and markers in the millions. GigaScience 8, giy154. doi: 10.1093/gigascience/giy154 30535326 PMC6365300

[B18] Ju C. Zhang W. Liu Y. Gao Y. Wang X. Yan J. . (2018). Genetic analysis of seedling root traits reveals the association of root trait with other agronomic traits in maize. BMC Plant Biol. 18, 171. doi: 10.1186/s12870-018-1383-5 30111287 PMC6094888

[B19] Lanctot A. (2022). Branching out underground: brassinosteroid signaling promotes lateral root development in rice. Plant Physiol. 189, 441–443. doi: 10.1093/plphys/kiac080 35212723 PMC9157082

[B20] Levene H. (1960). “ Robust tests for equality of variances,” in Contributions to Probability and Statistics. Ed. Olkin I. ( Stanford University Press, Stanford, CA), 278–292.

[B21] Li M. Zhang Y. W. Xiang Y. Liu M. H. Zhang Y. M. (2022b). IIIVmrMLM: The R and C++ tools associated with 3VmrMLM, a comprehensive GWAS method for dissecting quantitative traits. Mol. Plant 15, 1251–1253. doi: 10.1016/j.molp.2022.06.002 35684963

[B22] Li M. Zhang Y. W. Zhang Z. C. Xiang Y. Liu M. H. Zhou Y. H. . (2022a). A compressed variance component mixed model for detecting QTNs and QTN-by-environment and QTN-by-QTN interactions in genome-wide association studies. Mol. Plant 15, 630–650. doi: 10.1016/j.molp.2022.02.001 35202864

[B23] Lynch J. (1995). Root architecture and plant productivity. Plant Physiol. 109, 7–13. doi: 10.1104/pp.109.1.7 12228579 PMC157559

[B24] Manghwar H. Hussain A. Ali Q. Liu F. (2022). Brassinosteroids (BRs) role in plant development and coping with different stresses. IJMS 23, 1012. doi: 10.3390/ijms23031012 35162936 PMC8835148

[B25] Pace J. Gardner C. Romay C. Ganapathysubramanian B. Lubberstedt T. (2015). Genome-wide association analysis of seedling root development in maize (*Zea mays* L.). BMC Genomics 16, 47. doi: 10.1186/s12864-015-1226-9 25652714 PMC4326187

[B26] Pritchard J. K. Stephens M. Donnelly P. (2000). Inference of population structure using multilocus genotype data. Genetics 155, 945–959. doi: 10.1093/genetics/155.2.945 10835412 PMC1461096

[B27] Purcell S. Neale B. Todd-Brown K. Thomas L. Ferreira M. A. Bender D. . (2007). PLINK: A tool set for whole-genome association and population-based linkage analyses. Am. J. Hum. Genet. 81, 559–575. doi: 10.1086/519795 17701901 PMC1950838

[B28] Rabier C.-E. Barre P. Asp T. Charmet G. Mangin B. (2016). On the accuracy of genomic selection. PLoS One 11, e0156086. doi: 10.1371/journal.pone.0156086 27322178 PMC4913905

[B29] Ren W. Zhao L. Liang J. Wang L. Chen L. Li P. . (2022). Genome-wide dissection of changes in maize root system architecture during modern breeding. Nat. Plants 8, 1408–1422. doi: 10.1038/s41477-022-01274-z 36396706

[B30] Saengwilai P. Nord E. A. Chimungu J. G. Brown K. M. Lynch J. P. (2014). Root cortical aerenchyma enhances nitrogen acquisition from low-nitrogen soils in maize. Plant Physiol. 166, 726–735. doi: 10.1104/pp.114.241711 24891611 PMC4213101

[B31] Schlichting C. D. (1986). The evolution of phenotypic plasticity in plants. Annu. Rev. Ecol. Systematics 17, 667–693. doi: 10.1146/annurev.es.17.110186.00331541139587

[B32] Schneider H. M. Klein S. P. Hanlon M. T. Nord E. A. Kaeppler S. Brown K. M. . (2020). Genetic control of root architectural plasticity in maize. J. Exp. Bot. 71, 3185–3197. doi: 10.1093/jxb/eraa084 32080722 PMC7260711

[B33] Sha X. Guan H. Zhou Y. Su E. Guo J. Li Y. . (2023). Genetic dissection of crown root traits and their relationships with aboveground agronomic traits in maize. J. Integr. Agric. 22, 3394–3407. doi: 10.1016/j.jia.2023.04.02242574925

[B34] Song W. Wang B. Hauck A. L. Dong X. Li J. Lai J. (2016). Genetic dissection of maize seedling root system architecture traits using an ultra-high density bin-map and a recombinant inbred line population. J. Integr. Plant Biol. 58, 266–279. doi: 10.1111/jipb.12452 26593310 PMC5066741

[B35] Tibbs Cortes L. Zhang Z. Yu J. (2021). Status and prospects of genome-wide association studies in plants. Plant Genome 14, e20077. doi: 10.1002/tpg2.20077 33442955 PMC12806871

[B36] Trachsel S. Kaeppler S. M. Brown K. M. Lynch J. P. (2011). Shovelomics: High throughput phenotyping of maize (*Zea mays* L.) root architecture in the field. Plant Soil 341, 75–87. doi: 10.1007/s11104-010-0623-830311153

[B37] Wang S. Zhou D. (2017). Research on phenotypic plasticity in plants: An overview of history, current status, and development trends. Acta Ecol. Sin. 37, 8161–8169. doi: 10.5846/stxb201611242412

[B38] Wei Z. Li J. (2016). Brassinosteroids regulate root growth, development, and symbiosis. Mol. Plant 9, 86–100. doi: 10.1016/j.molp.2015.12.003 26700030

[B39] Wen T. J. Hochholdinger F. Sauer M. Bruce W. Schnable P. S. (2005). The roothairless1 gene of maize encodes a homolog of sec3, which is involved in polar exocytosis. Plant Physiol. 138, 1637–1643. doi: 10.1104/pp.105.062174 15980192 PMC1176433

[B40] Woll K. Borsuk L. A. Stransky H. Nettleton D. Schnable P. S. Hochholdinger F. (2005). Isolation, characterization, and pericycle-specific transcriptome analyses of the novel maize lateral and seminal root initiation mutant rum1. Plant Physiol. 139, 1255–1267. doi: 10.1104/pp.105.067330 16215225 PMC1283763

[B41] Wray N. R. Yang J. Hayes B. J. Price A. L. Goddard M. E. Visscher P. M. (2013). Pitfalls of predicting complex traits from SNPs. Nat. Rev. Genet. 14, 507–515. doi: 10.1038/nrg3457 23774735 PMC4096801

[B42] Wu B. Ren W. Zhao L. Li Q. Sun J. Chen F. . (2022). Genome-wide association study of root system architecture in maize. Genes (Basel) 13, 181. doi: 10.3390/genes13020181 35205226 PMC8872597

[B43] Xiao Y. Liu H. Wu L. Warburton M. Yan J. (2017). Genome-wide association studies in maize: Praise and stargaze. Mol. Plant 10, 359–374. doi: 10.1016/j.molp.2016.12.008 28039028

[B44] Yang J. Lee S. H. Goddard M. E. Visscher P. M. . (2011). GCTA: a tool for genome-wide complex trait analysis. AJHG 88, 76–82. doi: 10.1016/j.ajhg.2010.11.011 21167468 PMC3014363

[B45] Zeng T. Meng Z. Yue R. Lu S. Li W. Li W. . (2022). Genome wide association analysis for yield related traits in maize. BMC Plant Biol. 22, 449. doi: 10.1186/s12870-022-03812-5 36127632 PMC9490995

[B46] Zhang H. Kiani T. T. Chen H. Liu J. Chen X. (2025). Genome wide association analysis reveals multiple QTLs controlling root development in maize. J. Integr. Agric. 24, 1656–1670. doi: 10.1016/j.jia.2023.10.04042574925

[B47] Zurek P. R. Topp C. N. Benfey P. N. (2015). Quantitative trait locus mapping reveals regions of the maize genome controlling root system architecture. Plant Physiol. 167, 1487–1496. doi: 10.1104/pp.114.251751 25673779 PMC4378147

