# Supplementary material for: Genome-wide association study and genomic prediction of root system architecture in a diverse field-grown maize panel
Source: Front Plant Sci. 2026 Sep 15;17:1918922. doi: 10.3389/fpls.2026.1918922 (PMC13619920; doi:10.3389/fpls.2026.1918922)
Supplement: Supplementary file 1 [file SupplementaryFile1.docx]

**Supplementary Tables**

Table S1 The field trials information of the this study

| Environment | Location | Latitude | Longitude | Year | Planting date | Harvest date | Planting density (plants/ha) | Soil type | Soil texture | Soil depth (cm) |
| --- | --- | --- | --- | --- | --- | --- | --- | --- | --- | --- |
| HN_2017 | Ledong County, Hainan | 18.43° N | 108.87° E | 2017 | 2017/5/11 | 2018/20/3 | 60,000 | Lateritic soil | Sandy loam | 50 |
| XJ_2019 | Manas, Xinjiang, | 44.30° N | 86.22° E | 2019 | 2019/21/4 | 2017/21/9 | 97,500 | Calcareous soil | Loam | 50 |
| XJ_2020 | Manas, Xinjiang, | 44.30° N | 86.22° E | 2020 | 2020/25/4 | 2020/20/9 | 97,500 | Calcareous soil | Loam | 50 |
| XJ_2021 | Manas, Xinjiang, | 44.30° N | 86.22° E | 2021 | 2021/26/4 | 2021/18/9 | 97,500 | Calcareous soil | Loam | 50 |

Dates are presented in the format YYYY/ DD / MM

Table S2 Descriptive statistics of nine RSA traits and Heritability

| Traits | Min | Mean | Max | SD | CV(%) | kurtosis | skewness | Heritability |
| --- | --- | --- | --- | --- | --- | --- | --- | --- |
| BRW | 2.83 | 4.41 | 5.92 | 0.55 | 12.47 | -0.03 | 0.23 | 0.60 |
| Angle | 28.78 | 45.46 | 74.71 | 7.13 | 15.68 | 0.59 | 0.49 | 0.35 |
| Density | 0.92 | 2.21 | 4.08 | 0.67 | 30.31 | -0.65 | 0.20 | 0.50 |
| N1stBR | 9.25 | 14.99 | 33.42 | 2.57 | 17.14 | 6.44 | 1.12 | 0.67 |
| N2stBR | 5.92 | 12.67 | 32.25 | 2.71 | 21.38 | 6.70 | 1.19 | 0.70 |
| NLBR | 6.33 | 18.42 | 36.33 | 5.96 | 32.35 | 0.34 | 0.74 | 0.65 |
| NCR | 3.33 | 4.51 | 6.50 | 0.54 | 11.97 | 0.56 | 0.52 | 0.24 |
| NSR | 4.00 | 6.10 | 8.83 | 0.85 | 13.93 | 0.12 | 0.45 | NA |
| TR | 33.83 | 56.54 | 114.17 | 10.63 | 18.80 | 1.85 | 0.82 | 0.71 |

Descriptive statistics were based on the mean value of nine RSA traits cross four environments, SD: standard deviation; CV: Coefficients of variation;BRW: brace root whorls; N1stBR: number of brace root on the top whorl; N2ndBR: number of brace root on the second whorl; NLBR: number of the left brace root on other whorls; NCR: number of crown roots; NSR: number of seminal roots; TR: number of total roots. NA means the data is not available.

Table S3: SNPs and candidate genes significantly associated with environmental plasticity of RSA detected by BLINK

| SNP | Chr | Position^a^ | *P* value^b^ | MAF^c^ | FDR^d^ | Candidate gene(AGPv2)^e^ | Candidate gene  (AGPv4)^f^ | Annotation^g^ | PVE(%)^h^ | Trait |
| --- | --- | --- | --- | --- | --- | --- | --- | --- | --- | --- |
| AX-91382780 | 2 | 42482345 | 2.62E-09 | 0.16 | 0.00122 | GRMZM2G041356 | Zm00001d003434 | carbohydrate metabolic process | 4.83 | N2ndBR |
| AX-90556586 | 9 | 37540531 | 1.02E-09 | 0.08 | 0.00047 | GRMZM2G027958 | Zm00001d045785 | Leucine-rich repeat receptor-like serine/threonine/tyrosine-protein | 10.44 | NCR |
| AX-91438010 | 9 | 139518316 | 2.46E-08 | 0.32 | 0.00571 | GRMZM2G063875 | Zm00001d047800 | HSP20-like chaperones superfamily protein | 7.07 | NCR |
| AX-91744353 | 7 | 172204282 | 4.84E-08 | 0.26 | 0.00751 | GRMZM2G081682 | Zm00001d022490 | Xylose isomerase | 4.92 | NCR |
| AX-91157665 | 10 | 1879733 | 1.13E-08 | 0.31 | 0.00234 | GRMZM2G043150 | Zm00001d023260 | Heterogeneous nuclear ribonucleoprotein Q | 7.69 | BRW |
| AX-90704060 | 1 | 208244133 | 1.24E-08 | 0.44 | 0.00234 | GRMZM2G101245 | Zm00001d032069 | Root-specific kinase 1 | 5.92 | BRW |
| AX-91757481 | 8 | 65566789 | 1.51E-08 | 0.16 | 0.00234 | NA | NA | NA | 9.14 | BRW |
| AX-90782749 | 2 | 197826478 | 9.07E-08 | 0.12 | 0.01055 | AC233910.1_FG010 | NA | Serine/threonine-protein kinase SRPK | 4.30 | BRW |
| AX-90556586 | 9 | 37540531 | 3.69E-21 | 0.08 | 1.72E-15 | GRMZM2G027958 | Zm00001d045785 | Leucine-rich repeat receptor-like serine/threonine/tyrosine-protein | 18.66 | NSR |

a: Position in base pairs for the lead SNP according to version AGPv2 of the maize reference sequence; b: *p* value of significant SNPs associated with each trait; c: minor allele frequency of significant SNPs; d: False discover rate (FDR) control of significant SNPs; e: a plausible biological candidate gene in the locus or the nearest annotated gene to the lead SNP, gene ID were from B73 reference genome (AGPv2); f: gene IDs of candidate genes in B73 reference genome AGPv4 version; g: annotation of candidate genes based on maizeGDB; h: single SNP PVE (phenotypic variation explained by single SNP). NA means the data is not available in the databas

Table S4: SNPs and candidate genes significantly associated with nine RSA traits

| SNP | Chr | Position^a^ | P value^b^ | MAF^c^ | FDR^d^ | Candidate gene(AGPv2)^e^ | Candidate gene  (AGPv4)^f^ | Annotation^g^ | PVE(%)^h^ | Environment | Trait |
| --- | --- | --- | --- | --- | --- | --- | --- | --- | --- | --- | --- |
| AX-90651927 | 1 | 7965132 | 4.00E-08 | 0.30 | 0.01861 | GRMZM2G075701 | Zm00001d027548 | smt2 - sterol methyl transferase2 | 8.95 | XJ_2019 | Angle |
| AX-91740418 | 7 | 151171738 | 3.85E-11 | 0.01 | 1.79E-05 | GRMZM2G025242 | Zm00001d021574 | (laz1 - lazarus ortholog1 | 8.08 | XJ_2020 | Angle |
| AX-90918390 | 4 | 234573403 | 4.49E-10 | 0.07 | 0.00010 | GRMZM2G145978 | Zm00001d053711 | Calcium ion binding | 8.60 | XJ_2020 | Angle |
| AX-91716618 | 7 | 21144608 | 2.44E-09 | 0.07 | 0.00038 | GRMZM2G472096 | Zm00001d019214 | MADS-transcription factor 75 | 8.85 | XJ_2020 | Angle |
| AX-90631589 | 3 | 67888662 | 7.58E-09 | 0.32 | 0.00088 | GRMZM2G416322 | Zm00001d040823 | Cysteine-rich receptor-like protein kinase 10 | 4.02 | XJ_2020 | Angle |
| AX-90803740 | 3 | 37357494 | 2.62E-11 | 0.29 | 1.22E-05 | GRMZM2G132913 | Zm00001d040310 | Leucine-rich repeat domain、F-box domain | 3.13 | XJ_2021 | Angle |
| AX-91108257 | 8 | 150622772 | 1.60E-10 | 0.39 | 7.46E-05 | GRMZM2G163841 | Zm00001d011590 | Putative NAC domain transcription factor superfamily protein | 7.85 | XJ_2019 | Density |
| AX-91709578 | 6 | 152777349 | 3.91E-10 | 0.14 | 9.09E-05 | GRMZM2G075782 | Zm00001d038420 | RING-type E3 ubiquitin transferase | 1.34 | XJ_2019 | Density |
| AX-91704633 | 6 | 127112986 | 9.22E-10 | 0.08 | 0.00014 | GRMZM5G899390 | Zm00001d037601 | Serine/threonine-protein phosphatase | 4.26 | XJ_2019 | Density |
| AX-91171333 | 10 | 54625701 | 1.43E-10 | 0.20 | 6.63E-05 | NA | NA | NA | 4.70 | XJ_2020 | Density |
| AX-90731657 | 2 | 5932523 | 1.06E-07 | 0.32 | 0.02473 | GRMZM2G023037 | Zm00001d002099 | Putative cyclic nucleotide-gated ion channel 9 | 3.88 | XJ_2020 | Density |
| AX-90749639 | 2 | 72149009 | 5.08E-08 | 0.07 | 0.02360 | NA | NA | NA | 5.62 | XJ_2019 | N1stBR |
| AX-91056411 | 7 | 132805454 | 1.04E-09 | 0.28 | 0.000372073 | GRMZM2G111152 | Zm00001d020927 | Protein kinase domain-containing protein | 5.59 | XJ_2020 | N1stBR |
| AX-90665607 | 1 | 57107387 | 1.60E-09 | 0.26 | 0.00037 | GRMZM2G013588 | Zm00001d029096 | fanconi anemia group I-like protein | 8.42 | XJ_2020 | N1stBR |
| AX-90602057 | 2 | 220981833 | 1.21E-10 | 0.20 | 5.63E-05 | GRMZM2G026311 | Zm00001d006183 | Vacuolar protein sorting-associated protein | 3.80 | XJ_2021 | N1stBR |
| AX-91226067 | 6 | 27240435 | 1.34E-08 | 0.24 | 0.00311 | GRMZM2G075286 | Zm00001d035479 | Putative WAK family receptor-like protein kinase | 7.32 | XJ_2021 | N1stBR |
| AX-90627205 | 5 | 144314846 | 3.02E-08 | 0.43 | 0.00467 | GRMZM2G178356 | Zm00001d016160 | Potassium channel KAT1 | 2.06 | XJ_2021 | N1stBR |
| AX-90610002 | 2 | 4988962 | 7.13E-14 | 0.07 | 3.31E-08 | GRMZM2G081886 | Zm00001d002051 | D-3-phosphoglycerate dehydrogenase | 5.00 | HN_2017 | N2ndBR |
| AX-91116520 | 9 | 3857708 | 3.18E-10 | 0.43 | 7.40E-05 | GRMZM2G048243 | Zm00001d044814 | Protein kinase domain superfamily protein | 0.01 | HN_2017 | N2ndBR |
| AX-90660826 | 1 | 39800263 | 3.01E-08 | 0.25 | 0.00466 | GRMZM2G109885 | Zm00001d028597 | tRNA(guanine(26)-N(2))-dimethyltransferase | 5.45 | HN_2017 | N2ndBR |
| AX-91434412 | 1 | 195491000 | 5.97E-10 | 0.08 | 0.00028 | GRMZM2G043198 | Zm00001d031659 | Pyruvate dehydrogenase E1 component subunit beta | 2.61 | XJ_2019 | N2ndBR |
| AX-90665607 | 1 | 57107387 | 1.38E-10 | 0.26 | 6.41E-05 | GRMZM2G013588 | Zm00001d029096 | fanconi anemia group I-like protein | 8.83 | XJ_2020 | N2ndBR |
| AX-90600928 | 1 | 236917955 | 1.83E-08 | 0.04 | 0.00426 | GRMZM2G130904 | Zm00001d032854 | prx59 - peroxidase59) | 5.14 | XJ_2020 | N2ndBR |
| AX-91109909 | 8 | 156426823 | 5.36E-08 | 0.19 | 0.02493 | GRMZM2G341442 | Zm00001d011769 | Tetraspanin-6 | 6.33 | XJ_2021 | N2ndBR |
| AX-90556586 | 9 | 37540531 | 8.16E-34 | 0.08 | 3.80E-28 | GRMZM2G027958 | Zm00001d045785 | Leucine-rich repeat receptor-like serine/threonine/tyrosine-protein | 23.97 | XJ_2019 | NCR |
| AX-90562319 | 4 | 15298829 | 1.30E-08 | 0.27 | 0.00302 | GRMZM2G070011 | Zm00001d049113 | Vignain/Senescence-specific cysteine protease SAG12 | 1.31 | XJ_2019 | NCR |
| AX-90973644 | 5 | 202292728 | 2.10E-08 | 0.42 | 0.00326 | GRMZM2G164948 | Zm00001d017823 | Thioredoxin reductase | 4.77 | XJ_2019 | NCR |
| AX-91708716 | 6 | 148587733 | 3.37E-08 | 0.21 | 0.00391 | GRMZM2G033641 | Zm00001d038262 | Patellin-5 (Fragment) | 0.67 | XJ_2019 | NCR |
| AX-91797627 | 9 | 103069253 | 2.92E-09 | 0.17 | 0.00136 | GRMZM2G111711 | Zm00001d046805 | Putative WRKY DNA-binding domain superfamily protein | 5.63 | XJ_2019 | NLBR |
| AX-90604539 | 3 | 5984702 | 2.80E-08 | 0.49 | 0.00650 | GRMZM2G143723 | NA | Hypothetical protein | 4.43 | XJ_2019 | NLBR |
| AX-90761400 | 2 | 117773787 | 3.70E-11 | 0.43 | 1.72E-05 | GRMZM2G162056 |  | hagtf13 - GNAT-transcription factor 13 | 4.41 | XJ_2020 | NLBR |
| AX-91699404 | 6 | 99946338 | 1.09E-08 | 0.29 | 0.00254 | GRMZM2G071659 | Zm00001d036831 | E3 ubiquitin-protein ligase RGLG1 | 2.81 | XJ_2020 | NLBR |
| AX-91183165 | 10 | 99109460 | 8.00E-10 | 0.10 | 0.00037 | GRMZM2G143725 | Zm00001d025010 | Protein transport protein Sec24-like | 1.93 | XJ_2021 | NLBR |
| AX-90532253 | 1 | 239440080 | 7.74E-13 | 0.46 | 3.60E-07 | GRMZM5G813143 | Zm00001d032921 | NADP-dependent oxidoreductase domain | 6.32 | HN_2017 | BRW |
| AX-91545100 | 2 | 189840375 | 6.06E-13 | 0.10 | 2.82E-07 | GRMZM2G125934 | Zm00001d006027 | BZIP transcription factor family protein | 3.44 | XJ_2020 | BRW |
| AX-90660884 | 1 | 40039763 | 1.88E-11 | 0.09 | 4.37E-06 | GRMZM2G179432 | Zm00001d028601 | Haloacid dehalogenase-like hydrolase (HAD) superfamily protein | 11.07 | XJ_2020 | BRW |
| AX-91695224 | 6 | 75543908 | 3.24E-10 | 0.06 | 5.03E-05 | GRMZM5G835276 | Zm00001d036237 | GDSL esterase/lipase | 6.40 | XJ_2020 | BRW |
| AX-91512155 | 2 | 15686398 | 1.17E-09 | 0.37 | 0.00014 | NA | NA | NA | 7.36 | XJ_2020 | BRW |
| AX-90556586 | 9 | 37540531 | 8.44E-25 | 0.08 | 3.92E-19 | GRMZM2G027958 | Zm00001d045785 | Leucine-rich repeat receptor-like serine/threonine/tyrosine-protein | 15.34 | HN_2017 | NSR |
| AX-90881229 | 4 | 95681474 | 1.91E-12 | 0.13 | 4.44E-07 | GRMZM2G162840 | Zm00001d050583 | Trihelix transcription factor ASR3 | 1.53 | HN_2017 | NSR |
| AX-91502307 | 1 | 264873315 | 5.92E-10 | 0.16 | 9.17E-05 | GRMZM2G118003 |  | Beta-glucosidase 44 | 5.81 | HN_2017 | NSR |
| AX-90615919 | 3 | 66730548 | 9.08E-09 | 0.43 | 0.00105 | GRMZM2G095039 | Zm00001d040803 | Protein RALF-like 33 | 2.15 | HN_2017 | NSR |
| AX-90637108 | 9 | 76421437 | 3.17E-08 | 0.18 | 0.00257 | GRMZM5G812774 | Zm00001d046305 | BES transcription factor | 4.10 | HN_2017 | NSR |
| AX-90563078 | 1 | 73510051 | 3.32E-08 | 0.33 | 0.00257 | GRMZM2G067371 | Zm00001d029529 | Ninja-family protein AFP3 | 3.34 | HN_2017 | NSR |
| AX-90556586 | 9 | 37540531 | 2.68E-20 | 0.08 | 1.25E-14 | GRMZM2G027958 | Zm00001d045785 | Leucine-rich repeat receptor-like serine/threonine/tyrosine-protein | 31.53 | XJ_2019 | NSR |
| AX-90994887 | 6 | 69389490 | 1.27E-09 | 0.37 | 0.00023 | NA | NA | NA | 1.78 | XJ_2019 | NSR |
| AX-91170661 | 10 | 52373376 | 1.49E-09 | 0.42 | 0.00023 | GRMZM5G809546 | NA | NA | 1.74 | XJ_2019 | NSR |
| AX-91355589 | 7 | 22526569 | 4.95E-08 | 0.41 | 0.00575 | GRMZM2G096604 | Zm00001d019241 | Uncharacterized protein | 1.60 | XJ_2019 | NSR |
| AX-91401269 | 9 | 152252450 | 7.39E-10 | 0.37 | 0.00034 | GRMZM2G178072 | [Zm00001d048385](https://www.maizegdb.org/gene_center/gene/Zm00001d048385) | PHD finger protein ING | 14.91 | HN_2017 | TR |
| AX-91207392 | 1 | 239518679 | 8.87E-09 | 0.47 | 0.00206 | GRMZM2G010871 | [Zm00001d032923](https://www.maizegdb.org/gene_center/gene/Zm00001d032923) | Heat stress transcription factor A-6b | 6.60 | HN_2017 | TR |
| AX-90640909 | 3 | 2103961 | 6.10E-08 | 0.26 | 0.00946 | GRMZM2G099678 | [Zm00001d039301](https://www.maizegdb.org/gene_center/gene/Zm00001d039301) | NA | 0.65 | HN_2017 | TR |
| AX-90604539 | 3 | 5984702 | 7.87E-11 | 0.49 | 3.66E-05 | GRMZM2G143723 | NA | Hypothetical protein | 4.70 | XJ_2019 | TR |
| AX-90858743 | 4 | 11884563 | 1.55E-09 | 0.24 | 0.00036 | GRMZM2G017557 | [Zm00001d049020](https://www.maizegdb.org/gene_center/gene/Zm00001d049020) | rtp1 - root preferential1 | 12.07 | XJ_2019 | TR |
| AX-91511518 | 2 | 12351954 | 9.53E-09 | 0.23 | 0.00148 | GRMZM2G125669 | [Zm00001d002436](https://www.maizegdb.org/gene_center/gene/Zm00001d002436) | (aox2 - alternative oxidase2) | 7.91 | XJ_2019 | TR |
| AX-90761400 | 2 | 117773787 | 4.72E-13 | 0.43 | 2.20E-07 | GRMZM2G162056 | [Zm00001d004611](https://www.maizegdb.org/gene_center/gene/Zm00001d004611) | hagtf13 - GNAT-transcription factor 13 | 6.50 | XJ_2020 | TR |
| AX-90562523 | 2 | 2120469 | 1.64E-08 | 0.17 | 0.00382 | GRMZM2G040736 | Zm00001d001865 | cytokinin response regulator1 ) | 5.43 | XJ_2020 | TR |
| AX-91798419 | 9 | 107198796 | 5.21E-08 | 0.08 | 0.02425 | GRMZM2G172357 | [Zm00001d046900](https://www.maizegdb.org/gene_center/gene/Zm00001d046900) | Grx_C4-glutaredoxin subgroup I | 4.12 | XJ_2021 | TR |

a: Position in base pairs for the lead SNP according to version AGPv2 of the maize reference sequence; b: *P* value of significant SNPs associated with each trait; c: minor allele frequency of significant SNPs; d: False discover rate (FDR) control of significant SNPs; e: a plausible biological candidate gene in the locus or the nearest annotated gene to the lead SNP, gene ID were from B73 reference genome (AGPv2); f: gene IDs of candidate genes in B73 reference genome AGPv4 version; g: annotation of candidate genes based on maizeGDB; h: single SNP PVE (phenotypic variation explained by single SNP). NA means the data is not available in the database.
