# Supplementary material for: Genome-wide association study and genomic prediction of root system architecture in a diverse field-grown maize panel
Source: Front Plant Sci. 2026 Sep 15;17:1918922. doi: 10.3389/fpls.2026.1918922 (PMC13619920; doi:10.3389/fpls.2026.1918922)
Supplement: Supplementary file 2 [file SupplementaryFile2.docx]

**Supplementary Figures**

Fig. S1 Evanno Methods for detecting the true number of groups K from STRUCTURE results

Fig.S2 Distribution of nine root system architecture (RSA) traits across the association panel. Histograms with density curves are shown for Angle, Density, N1stBR, N2ndBR, NLBR, NCR, NSR, TR, and BRW. Density plot of nine RSA traits. BRW: brace root whorls; N1stBR: number of brace root on the top whorl; N2ndBR: number of brace root on the second whorl; NLBR: number of the remaining brace root on other whorls; NCR: number of crown roots; NSR: number of nodal/seminal roots; TR: number of total roots.

Fig.S3 Phenotypic variation and environmental plasticity of nine root system architecture (RSA) traits. (A) Boxplots show the distribution of each RSA trait across the four field environments (HN_2017, XJ_2019, XJ_2020, and XJ_2021). Pairwise P values from Games-Howell post-hoc tests indicate the significance of environmental differences. (B) Violin plots show the distribution of the CV-based environmental plasticity index for each trait across the association panel. Density, NLBR, and NSR exhibited greater environmental plasticity than the other measured RSA traits.

Fig.S4 Manhattan plots of significant main-effect quantitative trait nucleotides (QTNs) identified by the IIIVmrMLM multi-environment GWAS model for nine root system architecture (RSA) traits across four environments (HN_2017, XJ_2019, XJ_2020, XJ_2021). Each panel represents the genome-wide association results for an individual RSA trait. The x-axis shows the physical positions of SNPs across the 10 maize chromosomes, and the y-axis shows the −log10(P-value) of each association. The dashed horizontal line indicates the significance threshold determined by LOD threshold (LOD > 3.0) and genome wide threshold with Bonferroni correction. A total of 242 significant main-effect QTNs were identified across all nine traits.

Fig.S5 Manhattan plots of significant QTN-by-environment interaction (QEI) effects identified by the IIIVmrMLM model for nine RSA traits. Each panel shows the genome-wide QEI signals for an individual trait. The x-axis represents the physical SNP positions along the 10 maize chromosomes, and the y-axis represents the LOD score of each QEI effect. The dashed horizontal line indicates the significance threshold (LOD > 3 with Bonferroni correction). A total of 210 significant QEIs were detected across all nine traits, highlighting loci whose genetic effects vary substantially across the four field environments (HN_2017, XJ_2019, XJ_2020, XJ_2021).

Fig. S6 Overlap analysis between significant associations identified by the BLINK model and the IIIVmrMLM model. (A) Venn diagram showing the overlap of main-effect QTNs detected by IIIVmrMLM and significant SNPs identified by BLINK. Four shared loci were identified: AX-90761400 (associated with N2ndBR and NLBR), AX-91798419 (associated with N2ndBR), AX-90973644 (associated with NCR), and AX-91512155 (associated with BRW). (B) Venn diagram illustrating the overlap of QTN-by-environment interactions (QEIs) from IIIVmrMLM and significant SNPs from BLINK. Three overlapping loci were detected: AX-90556586 (associated with NCR, NSR, and BRW), AX-91512155 (associated with BRW), and AX-91511518 (associated with NLBR). These overlapping loci represent genetic variants with robust and reproducible associations across statistical models and environments.

Fig. S7 Additive (A) and dominance (B) effects of three overlapping SNPs with significant QTN-by-environment interaction (QEI) signals across four environments. These SNPs: AX-90556586, AX-91512155, and AX-91511518 were identified as significant in both BLINK single-environment GWAS and as QEIs in the IIIVmrMLM multi-environment analysis. AX-90556586 showed additive effects on brace root whorls (BRW), crown root number (NCR), and nodal/seminal root number (NSR); AX-91512155 on BRW; and AX-91511518 on the number of lower brace roots (NLBR). Environment abbreviations: HN_2017 (Hainan, 2017), XJ_2019 (Xinjiang, 2019), XJ_2020 (Xinjiang, 2020), XJ_2021 (Xinjiang, 2021). The reversal of effect direction and magnitude across environments illustrates the strong environment-dependency of these loci, consistent with their classification as QEIs rather than stable main-effect quantitative trait nucleotides.
